# Chromosome-level genome assembly and annotation of the black sea urchin *Arbacia lixula* (Linnaeus, 1758)

**DOI:** 10.1093/dnares/dsae020

**Published:** 2024-06-22

**Authors:** Carles Galià-Camps, Carlos Carreras, Marta Pascual, Carola Greve, Tilman Schell, Xavier Turon, Creu Palacín, Rocío Pérez-Portela, Owen S Wangensteen, Cinta Pegueroles

**Affiliations:** Department of Genetics, Microbiology and Statistics, Faculty of Biology, University of Barcelona, Barcelona, Spain; Institut de Recerca de la Biodiversitat (IRBio), Faculty of Biology, University of Barcelona, Barcelona, Spain; Department of Genetics, Microbiology and Statistics, Faculty of Biology, University of Barcelona, Barcelona, Spain; Institut de Recerca de la Biodiversitat (IRBio), Faculty of Biology, University of Barcelona, Barcelona, Spain; Department of Genetics, Microbiology and Statistics, Faculty of Biology, University of Barcelona, Barcelona, Spain; Institut de Recerca de la Biodiversitat (IRBio), Faculty of Biology, University of Barcelona, Barcelona, Spain; Centre for Translational Biodiversity Genomics (LOEWE-TBG), Frankfurt am Main, Germany; Senckenberg Forschungsinstitut und Naturmuseum, Senckenberganlage 25, 60325 Frankfurt am Main, Germany; Centre for Translational Biodiversity Genomics (LOEWE-TBG), Frankfurt am Main, Germany; Senckenberg Forschungsinstitut und Naturmuseum, Senckenberganlage 25, 60325 Frankfurt am Main, Germany; Department of Marine Ecology, Centre for Advanced Studies of Blanes, Spanish National Research Council (CEAB, CSIC), Blanes, Spain; Institut de Recerca de la Biodiversitat (IRBio), Faculty of Biology, University of Barcelona, Barcelona, Spain; Department of Evolutionary Biology, Ecology and Environmental Sciences, Faculty of Biology, University of Barcelona, Barcelona, Spain; Institut de Recerca de la Biodiversitat (IRBio), Faculty of Biology, University of Barcelona, Barcelona, Spain; Department of Evolutionary Biology, Ecology and Environmental Sciences, Faculty of Biology, University of Barcelona, Barcelona, Spain; Institut de Recerca de la Biodiversitat (IRBio), Faculty of Biology, University of Barcelona, Barcelona, Spain; Department of Evolutionary Biology, Ecology and Environmental Sciences, Faculty of Biology, University of Barcelona, Barcelona, Spain; Department of Genetics, Microbiology and Statistics, Faculty of Biology, University of Barcelona, Barcelona, Spain; Institut de Recerca de la Biodiversitat (IRBio), Faculty of Biology, University of Barcelona, Barcelona, Spain

**Keywords:** chromosome-level assembly, genome annotation, mitochondrial genome, sea urchin, *Arbacia lixula*

## Abstract

The black sea urchin (*Arbacia lixula*) is a keystone species inhabiting the coastal shallow waters of the Mediterranean Sea, which is a key driver of littoral communities’ structure. Here, we present the first genome assembly and annotation of this species, standing as the first Arbacioida genome, including both nuclear and mitochondrial genomes. To obtain a chromosome-level assembly, we used a combination of PacBio high fidelity (HiFi) reads and chromatin capture reads (Omni-C). In addition, we generated a high-quality nuclear annotation of both coding and non-coding genes, by using published RNA-Seq data from several individuals of *A. lixula* and gene models from closely related species. The nuclear genome assembly has a total span of 607.91 Mb, being consistent with its experimentally estimated genome size. The assembly contains 22 chromosome-scale scaffolds (96.52% of the total length), which coincides with its known karyotype. A total of 72,767 transcripts were predicted from the nuclear genome, 24,171 coding, and 48,596 non-coding that included lncRNA, snoRNA, and tRNAs. The circularized mitochondrial genome had 15,740 bp comprising 13 protein-coding genes, 2 rRNA, and 22 tRNA. This reference genome will enhance ongoing *A. lixula* studies and benefit the wider sea urchin scientific community.

## 1. Introduction

The black sea urchin, *Arbacia lixula*, is an iconic species living in the Mediterranean Sea and Atlantic Ocean (mainly in the Macaronesian Islands). Like other sea urchins, it is a key driver of littoral communities’ structure due to its contribution to maintaining marine barren grounds, shifting complex littoral macroalgal beds into systems dominated by crustose coralline algae.^1,2^ The effect of *A. lixula* is particularly pronounced given its omnivorous diet and bulldozing mode of browsing.^3,4^ Being a thermophilous species, it is expected to be favoured by climate change.^2,5,6^ Previous studies showed that increased seawater temperature seems to favour both gametes’ production and larval development and survival.^7–9^ In addition, a transcriptomic study found compelling evidence of phenotypic plasticity in this species and reported strong gene expression shifts in individuals exposed to acute thermal changes.^10^ Overall, it is suspected that it could displace other species less favoured by global warming such as *Paracentrotus lividus*.^11^ In fact, over the last few decades, it has become increasingly common in the coldest regions of the Mediterranean Sea.^8,12^ The potential spread of *A. lixula* may negatively affect the shallow rocky ecosystems along the Mediterranean coasts, as it has the potential to generate and maintain barrens of reduced diversity and productivity through massive grazing.^2,5^ Due to its ecological relevance and predicted increased impact, it is important to undertake population genetic studies on this species.^1^

Population genetic studies are essential to understand the biology of the species and to predict future scenarios driven by climate change, by assessing several factors such as the connectivity between populations, genetic diversity, and inbreeding levels, among others. Several studies have evaluated the population genetic structure of *A. lixula*, with different conclusions depending on the resolution level of the markers used.^6,13,14^ Genetic differentiation between the Mediterranean Sea and the Atlantic Ocean was reported regardless of the marker used. However, panmixia across the Mediterranean Sea could only be ruled out by moving from genetic markers (mitochondrial loci, microsatellites) to genomic data.^14^ The two main drivers of its genetic structure seemed to be salinity and, to a lesser degree, temperature. Additionally, more recent studies demonstrated the existence of strong genomic substructure along a naturally acidified system (pH from 8.1 to 7.4) over distances of less than 200 m and likely related to adaptation to pH.^15^ A recent study reported that only less than 3% of the genomic markers from *A. lixula*^14^ could be mapped to the closest reference genome available (*Strongylocentrotus purpuratus*), which was insufficient to characterize the functionality of candidate loci to be involved in adaptation.^16^ Thus, due to the lack of a reference genome for this species, the genetic bases of adaptation to these and other potential factors are still unknown.

From the evolutionary point of view, sea urchins are in a key position in the tree of life. As deuterostomes, echinoderms, and their sister group hemichordates (marine worms) are the only groups sharing a common ancestor with chordates. Thus, research on sea urchins encompasses several fields and applications, such as evolutionary biology, cell biology, biochemistry of eggs, embryos, and the fertilization process, and human diseases (reviewed in Davidson et al., 2002^17^). Currently, there are five nuclear reference genomes of sea urchins available: *S. purpuratus* (GCA_000002235.4),^18^*P. lividus* (GCA_940671915.1),^19^*Lytechinus variegatus* (GCA_018143015.1),^20^*Lytechinus pictus* (GCA_015342785.2),^21^ and *Eucidaris tribuloides* (GCA_001188425.1). However, the most closely related genomes diverged from *A. lixula* around 179 Mya (http://www.timetree.org/, accessed November 2023) which limits comparative genomic studies.^16^

Here, we provide the first high-quality genome assembly of *A. lixula*, including both nuclear and mitochondrial genomes. To achieve a robust assembly and annotation, we used a combination of sequencing techniques, including PacBio HiFi reads, Omni-C reads, and RNAseq short reads from several individuals. The nuclear genome is assembled at the chromosome level, the mitochondrial genome is circularized and both are annotated. The present genome will extend current genomic resources on sea urchins and will provide a key tool for assessing the population structure at a fine level and unravelling the adaptive capabilities of the species in the face of current challenges due to global warming and ocean acidification.

## 2. Materials and methods

### 2.1. Sampling, DNA extraction, library preparation, and sequencing

Two individuals of *A. lixula* were collected in Colera, Girona, Spain (42.394254, 3.154585). High molecular weight genomic DNA from muscle tissue from Aristotle’s lantern of a single individual was extracted according to the protocol of Sambrook and Russell (2001).^22^ The fragment length and concentration of the DNA extraction were assessed using the TapeStation 2200 (Agilent Technologies) and the Qubit dsDNA BR Reagents Assay Kit on the Qubit Fluorometer (Thermo Fisher Scientific, Waltham, MA, USA). Subsequently, we constructed two SMRTbell libraries following the instructions of the SMRTbell Express Prep kit 2.0 with low and ultra-low DNA Input Protocols (Pacific Biosciences, Menlo Park, CA, USA). We performed two SMRT cell sequencing runs from a single low-input library and a 3rd SMRT cell run of the ultra-low input library, and sequenced them in Continous Circular Sequence (CCS) mode on the Sequel System IIe with the Sequel II Sequencing Kit 2.0. To generate Omni-C short reads, ~50 mg of muscle tissue from the second individual was used by preparing the corresponding libraries following Dovetail® Omni-C kit manufacturer’s instructions with an insert size of 350 bp. The library was sequenced on the NovaSeq 6000 platform using a 150 paired-end sequencing strategy at Novogene (UK).

### 2.2. Genome size estimation

We estimated the genome size of *A. lixula* following the flow cytometry protocol with propidium iodide (PI)-stained nuclei described in Chueca et al., 2021.^23^ We selected muscle tissue from Aristotle’s lantern of the two freshly collected individuals of *A. lixula* mentioned above and chopped it with a razor blade in Petri dishes containing 2 ml of ice-cold Galbraith buffer.^24,25^ The suspension was filtered through a 42 μm nylon mesh, stained with the intercalating fluorochrome (PI, Thermo Fisher Scientific), and treated with RNase II A (Sigma–Aldrich), each with a final concentration of 25 μg/ml. The mean red PI fluorescence signal of stained nuclei was quantified using a Beckman–Coulter CytoFLEX flow cytometer with a solid-state laser emitting at 488 nm. Fluorescence intensities of 20,000 nuclei per sample were recorded. The software CytExpert 2.3 was used for histogram analyses. As internal reference standards, we used cricket (*Acheta domesticus*, 1C = 2 Gb) and chicken nuclei (*Gallus gallus*, 1C = 1.2 Gb) (Biosure). The total quantity of DNA in the sample was calculated as the ratio of the mean red fluorescence signal of the 2C peak of the stained nuclei of the *A. lixula* sample divided by the mean fluorescence signal of the 2C peak of the reference standard times the 1C amount of DNA in the reference standard. To minimize the instrument error, two measurements were carried out on each of three different days using both internal reference standards (Supplementary Table S1).

### 2.3. *De novo* genome assembly

High fidelity (HiFi) reads were called using a pipeline containing DeepConsensus 0.2.0.^26^ Briefly, all CCS reads are called from the subreads with PacBio’s tool ccs 6.4.0 (https://github.com/PacificBiosciences/ccs) followed by alignment of CCS and subreads with actc 0.3.1 (https://github.com/PacificBiosciences/actc) and finally running DeepConsensus with the before created CCS reads and alignment as input. Omni-C reads were first checked for quality using FastQC 0.11.9 (https://www.bioinformatics.babraham.ac.uk/projects/fastqc/) and subsequently trimmed to remove adapters and low-quality regions using Trimmomatic 0.39 (ILLUMINACLIP:TruSeq3-PE.fa:2:30:10 LEADING:3 TRAILING:3 SLIDINGWINDOW:4:15 MINLEN:36).^27^ The quality of the filtered reads was checked using FastQC. A primary screen of our potential genome assembly span and heterozygosity was obtained with the software Jellyfish 2.3.0^28^ and Genomescope 2.0.0^29^ using HiFi data and default parameters. HiFi reads from the PacBio platform were used to assemble the *A. lixula* genome using Hifiasm 0.16.1.^30^ Basic contiguity statistics of these assemblies were estimated using Quast 5.0.2^31^ (Supplementary Table S2). Duplicated regions (haplotigs) from the primary assembly were collapsed using purge_dups 1.2.5.^32^

### 2.4. Chromosome level assembly and mitochondrial identification

Filtered Omni-C data was used for chromosome scaffolding with YaHS 1.2a.2.^33^ The contact map of the obtained assembly was visualized and manually curated using Juicebox Assembly Tools,^34^ resulting in an assembly including chromosome-scale superscaffolds. Scaffolds were sorted by length, the largest 22 were numbered ascending from 1 to 22, most likely representing the 22 expected chromosomes.

The mitochondrial genome was obtained by MitoHiFi 2.2.^35^ A complete mitochondrial genome from *A. lixula* (obtained from NCBI X80396) was provided as input together with the manually curated scaffolds. The scaffold containing the mitochondrial genome was tagged accordingly in the final fasta file.

### 2.5. Assembly quality

The quality of each assembly stage was assessed using the following parameters: assembly contiguity, assembly completeness, and mapping rates. Assembly contiguity was assessed with Quast (Supplementary Table S2). Assembly completeness was measured by Benchmarking Universal Single-Copy Orthologs (BUSCO) 5.4.3^36^ against a Metazoan database (metazoan_odb10; Supplementary Table S3). We also evaluated the k-mer completeness of our assembly using Merqury 1.3.^37^ We identified the unplaced reads using blastn searches against the nt database, using the megablast engine with -evalue 1e-25 -max_target_seqs 1 -max_hsps 1 options (Supplementary Table S4). Finally, HiFi reads were back-mapped to the different assembly stages using minimap2 2.24,^38^ and heterozygosity levels were calculated after base-calling with bcftools 1.13.^39^ Results were summarized using Qualimap 2.2.1 (Supplementary Table S5).^40^

### 2.6. Repeated elements identification and masking

Repeat families were identified using RepeatModeler 2.0.4 and masked with RepeatMasker 4.1.4.^41^ First, we generated custom repeat libraries (CRL) from *A. lixula* and *P. lividus* by running RepeatModeler. We combined this library with the repeat sequences of *S. purpuratus* from RepBase 27.03,^42^ generating a final file containing repeat models for three genomes of sea urchins (*A. lixula*, *P. lividus*, and *S. purpuratus*). We soft-masked and hard-masked the repeats in the final assembly using RepeatMasker. Finally, we screened the genome assembly for telomeric repetitive motifs with the software tidk 0.2.31.^43^ We explored the most abundant repetitive elements with a size from 10 to 40 in the terminal 1% of each chromosome using the function ‘explore’ and, afterwards, we calculated the number of times they were repeated along the assembly using 1,000 bp windows with the function ‘search’.

### 2.7. Genome annotation

The annotation of protein-coding genes, small nucleolar RNA (snoRNAs), tRNAs, and UTR regions was performed using a combination of homology-based and *ab initio* methods with MAKER 2.31.10.^44^ For the homology-based methods, we used RNAseq from *A. lixula* and gene annotations from closely related species. Published RNA-seq data from *A. lixula* was downloaded from the SRA archive (PRJNA642021 and PRJNA302797) and consisted of 26 paired libraries with Illumina sequencing (Supplementary Table S6) from four different tissues: coelomocytes, digestive, ovary, and testis.^45^ Quality checking was performed using FastQC 0.11.9 and MultiQC 1.8^46^ with default parameters. Adapters and low-quality regions were removed using Trimmomatic v0.39 (TruSeq3-PE.fa:2:30:10 LEADING:3 TRAILING:3 SLIDINGWINDOW:4:15 MINLEN:36). In addition, we detected and removed contaminants (mainly human and bacterial sequences) with Kraken 2.1.2.^47^ Filtered RNAseq data was assembled with Trinity 2.11^48^ and redundancy was reduced using CD-HIT 4.8^49^ with a 90% similarity threshold. Additionally, all protein sequences from *S. purpuratus* and *P. lividus* annotations were downloaded (38,349 proteins from GCF_000002235.5 and 30,556 from Marlétaz et al., 2023^19^) and obtained amino acid sequences of BUSCO genes, localized in the newly generated genome assembly of *A. lixula*. We used these annotations in *A. lixula*, *S. purpuratus*, and *P. lividus* as input data to generate homology-based gene predictions on the hard-masked genome. To do so, we used BLASTn 2.10.0^50^ and exonerate 2.4.0 (https://www.ebi.ac.uk/about/vertebrate-genomics/software/exonerate) as implemented in MAKER 2.31.10.^51^ In addition, we conducted *ab initio* gene predictions with three different software: AUGUSTUS 3.3.3,^52^ GeneMark-EP 4.69,^53^ and SNAP 2013-11-29.^54^ To refine the genome annotation, we performed a total of three rounds of protein modelling. For the first modelling round, we used BUSCO genes to generate a gene model with SNAP and RNAseq for AUGUSTUS. Subsequently, we generated the first annotation draft with MAKER. For the 2nd and 3rd modelling rounds, we trained AUGUSTUS and created a new SNAP model with gene models of the previous round. Additionally, in the last annotation round, we also used tRNAscan-SE 1.3.1^55^ to annotate tRNA genes and snoscan 0.9.1^56^ to annotate snoRNA. Genes and transcripts annotated with MAKER were renamed using maker_map_ids and map_gff_ids scripts from MAKER 2.31.10. Protein coding, snoRNA, and tRNA genes were named ALIXG[0-9]{6} and their transcripts ALIXT[0-9]{6}-T[0-9]. Finally, to evaluate the completeness of the annotated protein-coding transcripts we ran BUSCO using the metazoa_odb10 database.

Annotation of long non-coding RNAs (lncRNAs), was conducted by mapping the filtered RNAseq reads to the reference genome using HISAT2 2.2.1121^57^ and generating a transcriptome assembly for each sample using StringTie 2.1.4122^58^ by providing the MAKER annotations as reference. The individual GTF files were merged to obtain a reference GTF using the merge option from StringTie, which was subsequently used as input for FEELnc^59^ to identify the candidate lncRNAs. Transcripts shorter than 200 bp, monoexonic, overlapping protein-coding genes, and transcripts with coding potential were filtered out. To compute the coding potential of the transcripts we used two different strategies to obtain the training sets: (i) the shuffle approach, which takes a set of mRNAs from *A. lixula* and shuffles them while preserving 7-mer frequencies, and (ii) using lncRNAs annotated in *S. purpuratus* as training set. A total of 23,344 non-coding transcripts (10,814 genes) in the shuffle approach and 22,049 non-coding transcripts (10,435 genes) were identified using the lncRNAs annotated in *S. purpuratus*. We selected the transcripts identified as non-coding by the two approaches (20,869 transcripts contained in 9,871 genes) and used them as input to estimate the coding potential using CPC2 (http://cpc2.gao-lab.org/, accessed on June 2023). CPC2 identified a total of 20,328 transcripts (9,627 genes) as non-coding, being our final set of putative lncRNAs. LncRNA genes were renamed using maker_map_ids as MSTRG.[0-9]{5} and transcripts as MSTRG.[0-9]{6}.[0-9]. Both MAKER and lncRNA annotations were combined to generate a final annotation GTF file for *A. lixula*.

Finally, the circularized mitochondrial genome was annotated using MITOS2 software (http://mitos2.bioinf.uni-leipzig.de/index.py, accessed in December 2022). Annotations were manually curated using Geneious Prime 2021.0.3 and compared to the five sea urchins mitochondrial genomes available: *Allocentrotus fragilis* (KC898200), *L. variegatus* (NC_037785), *P. lividus* (J04815), and *S. purpuratus* (NC_001453). Divergence times (in Myr) were obtained from TimeTree (http://www.timetree.org/) with the only exception of S. *purpuratus* and *A. fragilis*, estimated in 7.2–14 MYA.^60,61^

## 3. Results and discussion

### 3.1. Nuclear genome assembly

**Figure 1.**
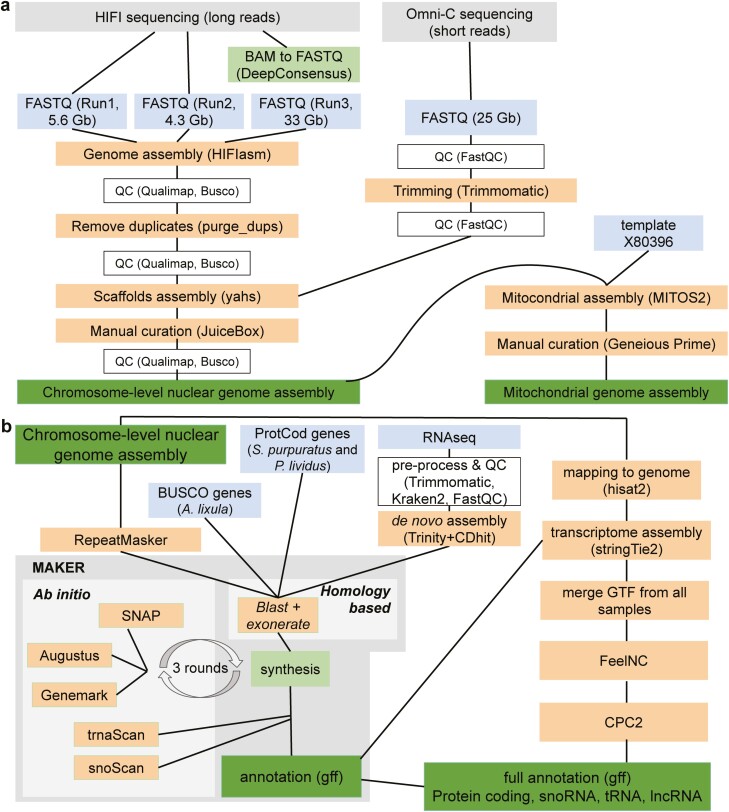
Workflow for (**a**) genome assembly and (**b**) annotation. Grey designates the sequencing data generated in the current research and available in ENA (PRJEB60287), blue designates input data, with squares the quality control steps, orange the processes and/or the software used, and dark green the output files generated.

**Figure 2.**
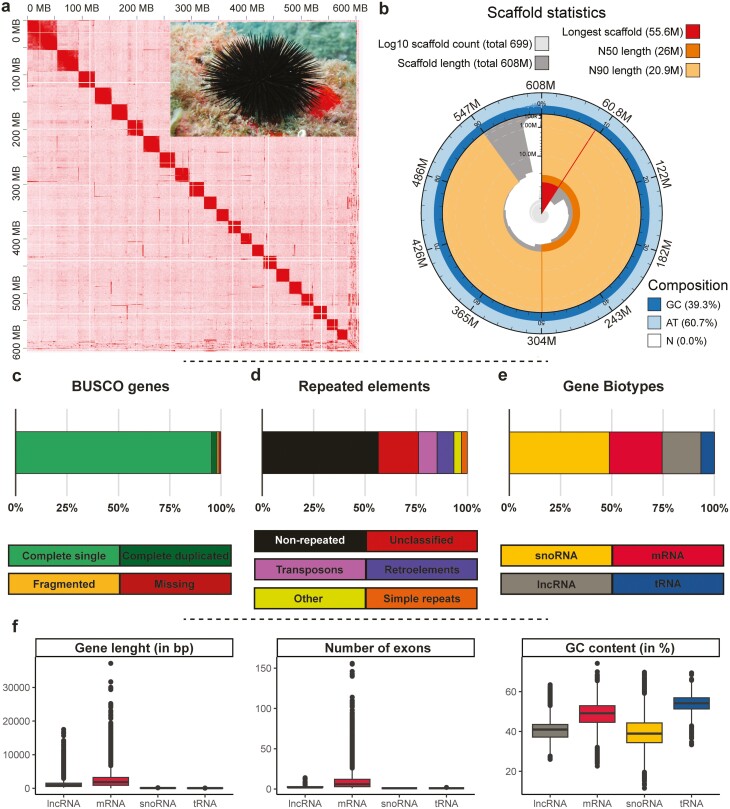
Nuclear genome assembly and annotation. (**a**) contact map of the genome assembly showing the 22 superscaffolds (corresponding to chromosomes) ordered from longest to shortest and a picture of *A. lixula* from Creu Palacín. (**b**) Snailplot summarizing scaffold statistics, total size, and base composition. The main plot is divided into ordered-size bins; pale grey spiral shows the cumulative length on a log scale, dark grey represents the length of all scaffolds, red represents the longest scaffold in the assembly (55.6 Mb), and orange and pale orange show the N50 and N90 lengths (26 Mb and 20.9 Mb), respectively. The dark blue and light blue around the outside of the plot show the distribution of GC and AT percentages. (**c**) Summary of the BUSCO genes identified using the metazoa_odb10 set. (**d**) Summary of the repeated elements identified in the genome assembly. (**e**) Classification of the genes annotated in the genome in major biotypes. (**f**) Gene length, number of exons, and percentage of GC content of the different gene biotypes.

The final genome assembly of *A. lixula* (eeArbLixu1) was generated by assembling 43 Gb of HiFi and 25 Gb of Omni-C data followed by manual curation, spanning 607.91 Mb (**Fig. 1a**). This assembly size is consistent with the genome size estimated experimentally using flow cytometry (580.21 Mb, Supplementary Table S1), but higher than when using the HiFi k-mer count distribution approach obtained with Genomescope (463.62 Mb, Supplementary Fig. S1). Differences in length between the total assembly and the flow cytometry are small (4.6%) and could be attributed to the variance in the experimental measurements, or to very small amounts of undetected haplotypic duplications and/or contamination in the assembly. The genome size inferred with K-mer-based estimates is likely underestimated due to the high amount of repeated elements and high heterozygosity levels (see below). The final unphased genome assembly is highly contiguous and complete (Supplementary Tables S2 and S3, **Fig. 2**). Importantly, 96.52% of the sequence is assembled in the 22 longest scaffolds, which agrees with the karyotype of the species (1n = 22; where *A. lixula* is called *A. pustulosa*^62^). The 676 unplaced scaffolds (representing 3.5% of the PacBio sequences) were taxonomically assigned using blast searches (Supplementary Table S4). A total of 50.44% of them had a significant match (with only 2% of mean query cover), where 87.98% were classified as Deuterostome, 6.74% as Protostome, 3.23% as Bacteria, and 2.05% as other (mostly synthetic sequences). Since 94.67% of the Deuterostome sequences were assigned to Echinodermata, we can conclude that roughly 50% of the unplaced scaffolds are likely to belong to *A. lixula*.

More than 99% of HiFi reads were back-mapped to the final assembly (Supplementary Table S5). The heterozygosity of the genome assembly with both back-mapping and HiFi k-mer count distribution is high (3.47 and 4.40%, respectively), in line with high values detected in reference genomes of other sea urchins, ranging from ~3% in *L. variegatus* to ~5% in *S. purpuratus*^63^ and with high heterozygosity levels detected in a previous population study in *A. lixula*.^14^ In the final assembly, we identified 98.01% (97.43% of them single-copy) complete BUSCOs and assembly k-mer completeness is 68.09%, possibly due to the high heterozygosity levels found in *A. lixula*. The percentage of GC content of the assembly is 39.33, higher than the other five sequenced sea urchins (36.26% on average, ranging from 34.4% in *P. lividus* to 37.40% in *S. purpuratus*, Supplementary Table S7). The lengths of all chromosomes, the mitochondrial genome, and unplaced scaffolds length are shown in Supplementary Table S8.

In the final assembly, 41.84% of its total length was identified as repeat elements, where 37.95% are interspersed repeats (Supplementary Table S9). This value is within the range of other sea urchins, being 41.02% on average, ranging from 39.20% in *S. purpuratus* to 42.68% in *L. pictus* (Supplementary Table S7). The most abundant elements were DNA transposons (9.16%), retroelements (8.06%), and simple repeats (2.99%), while most of the repeated elements remained unclassified (19.53%). A high abundance of DNA transposons followed by retrotransposons is usually found in sea urchins and other deuterostomes.^19^ We found several different numbers of repeats for the motif AACCCT as potential indicative for telomeric regions (Supplementary Fig. S2). The single motif was sparsely found along the genome but the six times tandem was found in high density in the terminal position of most chromosomes, indicating telomeric regions. We also found high-density regions in the middle of two chromosomes, indicating centromeric regions. The same motif being repeated in tandem three times was identified as telomeric in the two sea urchins with putative telomeric regions annotated to date (*Echinus esculentus* and *Psammechinus miliaris*, https://github.com/tolkit/a-telomeric-repeat-database). Our results point out that the telomeric motif in *A. lixula* is the six-time repeated tandem, supposing the longest telomeric repetitive element reported so far in sea urchins.

### 3.2. Nuclear genome annotation

Complete genome annotations are generally required to decipher functional aspects of many biological fundamental questions. Their obtention is challenging and computationally demanding due to its complexity, and given the tissue (and even cell) gene expression specificity, there is a need for sequencing transcriptomes from different individuals, development stages, conditions, and tissues. Thus, we annotated the *A. lixula* genome using RNAseq data from several tissues and individuals and annotations from closely related species, combining them using *ab initio* and homology-based gene prediction methods (**Fig. 1b**). Overall, a total of 19,415 protein-coding genes and 24,171 transcripts (1.24 transcripts per gene on average, with a maximum of 16 transcripts per gene) were annotated (**Table 1**). This number is within the range of other sea urchins, ranging from 1.47 to 1.12 in *L. variegatus* and *L. pictus*, respectively (Supplementary Table S7). However, this number might be an underestimation of the biological number of isoforms, and further studies adding additional individuals, tissues, and/or stages are advised to improve the current annotation. Protein-coding transcripts contain nine exons on average (Supplementary Table S10), with 3.6% of them being mono-exonic (874 out of 24,171 transcripts). The protein set resulting from annotation has a BUSCO completeness of 88.78%, being slightly lower than the BUSCO completeness recovered in the total assembly (98.01%). This indicates that despite the large number of annotated protein-coding genes a small fraction of genes is still missing in the annotation but present in the assembly. In addition, we annotated 37,895 non-coding genes, including 9,627 lncRNA (20,328 transcripts), 24,875 snoRNA, and 3,393 tRNAs (Supplementary Table S7).

**Table 1.** Elements annotated in *A. lixula* genome.

Element	Number
mRNA	19,415 (24,171)
lncRNA	9,627 (20,328)
snoRNA	24,875
tRNA	3,393
CDS	200,894
exon	244,694
five_prime_UTR	18,823
three_prime_UTR	26,293

For mRNA and lncRNA, numbers outside parentheses refer to genes and inside parentheses to transcripts.

### 3.3. Mitochondrial genome assembly and annotation

**Figure 3.**
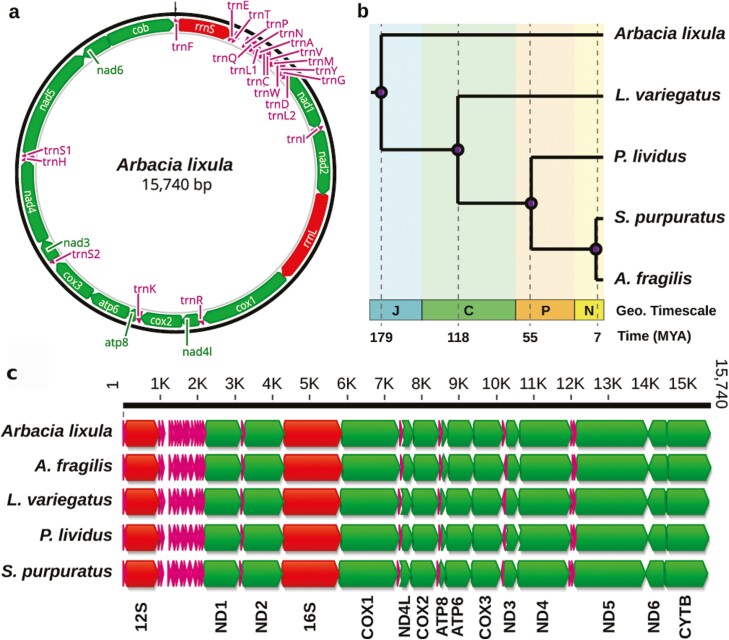
Mitochondrial genome assembly and annotation. (**a**) Circular plot showing the complete annotation of the *A. lixula* mitochondrial genome, including 13 protein-coding genes, 2 ribosomal RNAs (rRNA), and 22 tRNAs. (**b**) Divergence time among the five sea urchins mitochondrial genomes available; J: Jurassic, C: Cretaceous, P: paleogene, and N: Neogene. (**c**) Alignment of the five mitochondrial genomes from sea urchins shows a high degree of conservation among genes and their order.

The mitochondrial genome was assembled in one single circular contig of 15,740 bp and had a GC content of 37.4%. Its annotation contained 13 protein-coding genes, 2 ribosomal RNAs, and 22 tRNAs (**Fig. 3**), consistent with the available mitochondrial genome from *A. lixula*.^64^ Gene order and number of genes are highly conserved among sea urchins, despite the most distant species analyzed diverged ~179 Mya (**Fig. 3**). Our new mitogenome assembly is complete and contributes to enriching the sequence diversity available.^65,66^

## 4. Conclusion

We provide the annotated genome of the black sea urchin *A. lixula*, which has been sequenced within the framework of the Catalan Initiative for the Earth Biogenome Project.^67^ This is the first chromosome-level reference for this order (Arbacioida) and will contribute to enriching the genome resources of sea urchins in particular, and biodiversity in general. In fact, this species diverged around the lower Jurassic (~179 Mya) from the closest sequenced sea urchin genomes. Given its key phylogenetic position and its high quality (in both assembly and annotation aspects), the *A. lixula* genome sequence will represent a valuable resource for the scientific community. The resource will facilitate deciphering its population structure dynamics and the genetic basis of adaptation which is key for the potential increase in a number of this ecologically relevant species.

## Supplementary Material

dsae020_suppl_Supplementary_Tables_S1-S10

dsae020_suppl_Supplementary_Figures_S1-S2

## Data Availability

All the sequencing data generated in this study, the nuclear and mitochondrial genome assemblies are available at the European Nucleotide Archive (https://www.ebi.ac.uk/ena/browser/home) under the BioProject with accession number PRJEB60287. Both nuclear and mitochondrial genome annotations are available at https://github.com/EvolutionaryGenetics-UB-CEAB/Arbacia_lixula_genome/.
