# Supplementary material for: Chromosome-level genome assembly and annotation of the black sea urchin Arbacia lixula (Linnaeus, 1758)
Source: DNA Res. 2024 Jun 22;31(4):dsae020. doi: 10.1093/dnares/dsae020 (PMC11310861; doi:10.1093/dnares/dsae020)

**Supplementary Figure Legends**

**Figure S1**: K-mer analysis for estimating the genome size of *A. lixula* using Genomescope.


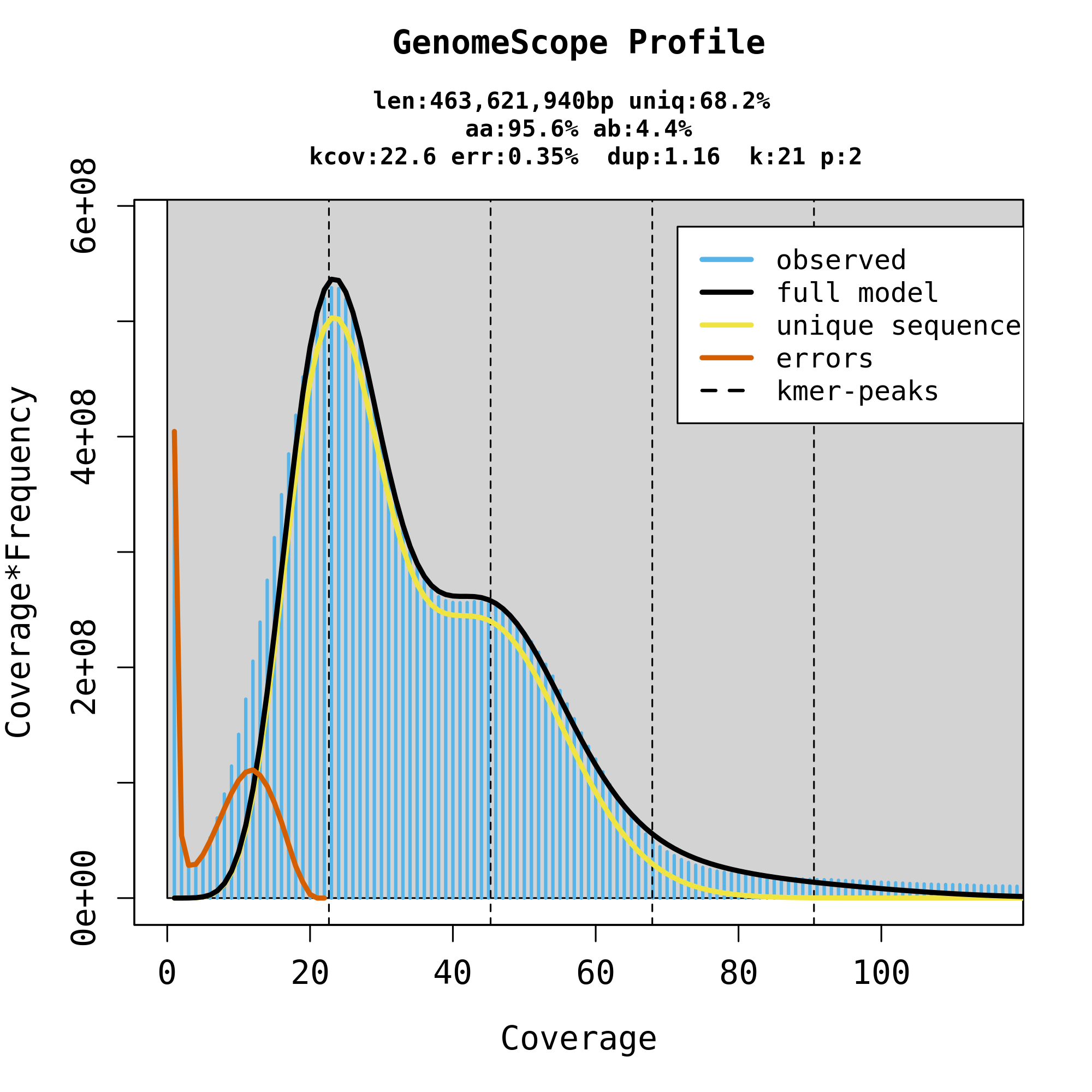


**Figure S2:** Distribution and abundance of the telomeric motif (the 6-time tandem repeated motif AATTTC, in black) and the single repeat unit (in blue). X axis represents the chromosome position (in bp) and in the Y axes the number of repeats found.


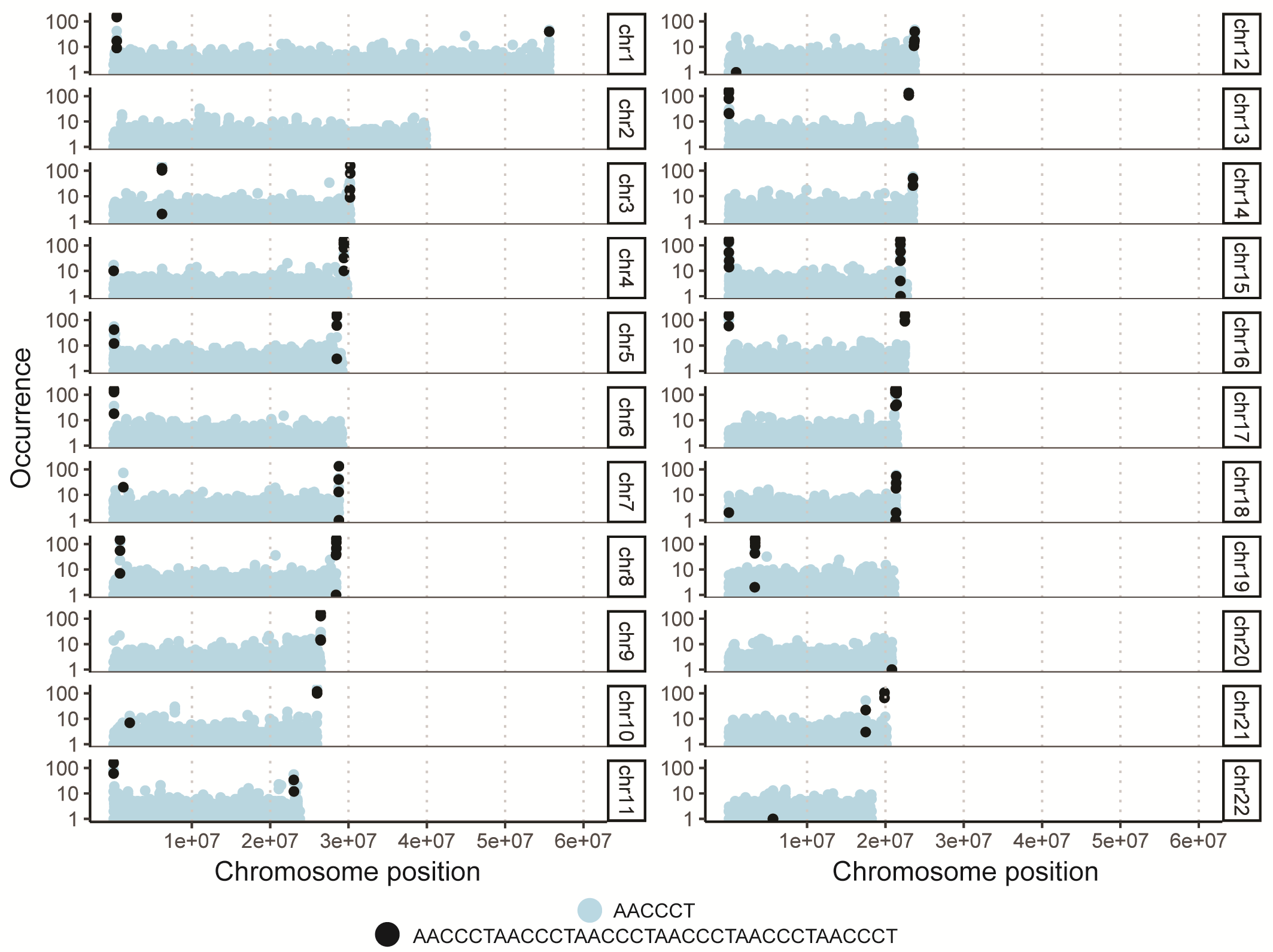

Supplement: dsae020_suppl_Supplementary_Figures_S1-S2 [file dsae020_suppl_supplementary_figures_s1-s2.docx]
